# Supplementary material for: Effects of preharvest sprays of iodine, selenium and calcium on apple biofortification and their quality and storability
Source: PLoS One. 2023 Mar 9;18(3):e0282873. doi: 10.1371/journal.pone.0282873 (PMC9997897; doi:10.1371/journal.pone.0282873)
Supplement: Attachment — Submitted filename: Response to Reviewers.docx [file pone.0282873.s001.docx]

We would like to thank the Reviewers for their kind words and pertinent comments, which have allowed improving the manuscript. A detailed point-by-point answers to the comments are provided below.

According to the requirements of PLOS ONE:

- we have added information in the Method section about permits received related to conducting field study and performing analyses/measurements (lines 90-94).

- we have removed in our manuscript the phrase “data not shown” for fruit firmness at harvest (line 324) and the data of this parameter have been presented in Table 4.

- we have checked the reference list and cited papers in the text. We have added also 3 literature items added (17, 18, 22). For that reason, we had to change the numbering of papers in both the reference list and text.

**Reviewer #1:**

**1)** As suggested by the Reviewer, we should add information about time of the day when the tested spray treatments were applied. However, we would like to state that the above information was given in “The tested spray treatments and trial setup” (line 179)

**2)** As suggested by the Reviewer, we should add an overview picture showing the tested treatments and results obtained. It seems that this suggestion is not justified because, in our opinion, the set-up of the experiment used is not complicated and the results presented in the tables and their description in the Results and Discussion sections are clear.

**3)** We agree with the Reviewer's argument to remove the phrase “with those nutrients” from Abstract (line 25).

**Reviewer #2:**

**1)** As suggested by the Reviewer, we have added in the Introduction section information about studies related to biofortification of tree fruits with iodine and selenium (lines 64-69).

**2)** Accordingly to the Reviewer’s suggestion, we have added information about pesticides and herbicides used and their application time (lines 130-138, 140-144).

**3)** As proposed by the Reviewer, we have added information that the control trees were not sprayed with water (lines 171-172).

**4)** As suggested by the Reviewer, in the Result section, we should explain the results of all treatments whereas in the Discussion section, we should highlight the reasons of their result findings in the light of available literature. However, the Reviewer did not indicate which parts in the sections of Results and Discussion require the improvement. According to our opinion, in the Results section, we precisely described the impacts of the tested spray treatments on individual characteristics/parameters of plants. Moreover, in the Discussion section, we confronted the results obtained with those of the available scientific literature.
